# A novel fusion of *SQSTM1* and *FGFR1* in a patient with acute myelomonocytic leukemia with t(5;8)(q35;p11) translocation

**DOI:** 10.1038/bcj.2014.86

**Published:** 2014-12-12

**Authors:** Y Nakamura, Y Ito, N Wakimoto, E Kakegawa, Y Uchida, M Bessho

**Affiliations:** 1 Department of Hematology, Saitama Medical University, Saitama, Japan

Hematological malignancies with *FGFR1* abnormality (8p11 myeloproliferative syndrome; EMS) are rare atypical stem cell disorders characterized by eosinophilia, T-cell proliferation and progression to acute myeloid leukemia.^1, 2^ In EMS, fibroblast growth factor receptor 1 (*FGFR1*) gene at 8p11 is disrupted by chromosomal translocation, resulting in the formation of chimeric products with various partner genes. To date, 13 *FGFR1* partners have been identified: *ZMYM2* (*ZNF198*) at 13q12; *CNTR* at 9q33; *FGFR1OP* at 6q27; *BCR* at 22q11; *HERVK* at 19p13.3; *FGFR1OP2* at 12p11; *TRIM24* (*TIF1*) at 7q34; *CPSF6* at 12q15; *MYO18A* at 17q23; *LRRFIP1* at 2q37; *CUX1* at 7q34; *TPR* at 1q25; and *RANBP2/NUP358* at 2q12.^2, 3, 4, 5^ In all reported cases in which chimeric transcripts were cloned, N-terminal portion of the predicted fusion protein is composed of a partner gene with a dimerization domain, which may induce the constitutive activation of FGFR1 tyrosine kinase in the C-terminal portion, leading to the cellular transformation.^2^

Here, we report the identification of sequestosome 1 (*SQSTM1*) gene at 5q35 as a novel fusion partner of the *FGFR1* in a patient with acute myelomonocytic leukemia presenting 8p11 chromosomal abnormality.

A 76-year-old man was referred to our hospital for anemia with hemoglobin level of 5.0 g/dl. The white blood cell count was 4.1 × 10^9^ cells/l with 11% blasts, 1% eosinophils, 29% neutrophils and 30% monocytes, Bone marrow (BM) was hypercellular with 43% blasts and 17% monocytes. Dysplastic changes were observed in peripheral neutrophils and BM megakaryocytes. Immunphenotyping revealed that leukemic cells were positive for CD13, CD33, CD34 and HLA-DR and negative for other myeloid and lymphoid markers. He was diagnosed as acute myelomonocytic leukemia and received blood transfusion therapy for anemia, but chemotherapy was not done because of poor performance status. He died of progressive disease 15 months after the diagnosis.

Chromosome analysis of the BM cells showed the karyotype as 46,XY,t(5;8)(q35;p11) (20/20 cells) (Figure 1a). To characterize the 8p11 translocation, Southern blot analysis of DNA from the patient's peripheral leukemic cells was performed using a genomic probe containing *FGFR1* exon 9 and 10, as chromosomal breaks occur within intron 8 in most cases with EMS. Abnormal bands were observed in sample with *Bam*HI or *Hin*dIII digestion (Figure 1b), confirming that *FGFR1* was rearranged by the translocation.

To determine the *FGFR1* partner gene, we searched for a *FGFR1* chimeric transcript by 5′-rapid amplification of cDNA end (RACE) method using 5'-Full RACE core set (Takara Bio, Otsu, Japan). Total RNA was extracted from the leukemic cells and the first cDNA strand was synthesized with phosphorylated primer in *FGFR1* exon 12 (5′-GCCAGATACTCCATGCCTCG-3′) by reverse transcription. After ligation, cDNA was amplified by PCR with *FGFR1*-10R1 (5′-ACACCACCTGCCCAAAGCAGC-3′) and *FGFR1*-10F1 (5′-AGGCTATCGGGCTGGACAAGG-3′) primers in exon 10. The PCR product was further amplified with *FGFR1*-10R2 (5′-CAGGGGTTTGCCTAAGACCAG-3′) and *FGFR1*-10F2 (5′-CGTGTGACCAAAGTGGCTGTG-3′) primes in exon 10. An abnormal band was amplified and sequencing of the PCR product revealed that *FGFR1* was fused to a 5′ foreign sequence that was identified as exon 9 of *SQSTM1* at chromosome 5q35, showing that *SQSTM1* was juxtaposed to *FGFR1* as the result of the chromosomal translocation.

To ascertain the formation of chimeric transcript, we performed reverse transcription PCR (RT-PCR) analysis, using *SQSTM1*-8F1 (5′-CCTGAAGAACGTTGGGAGAG-3′) for exon 8 and *FGFR1*-10R1 primers. An amplified product was obtained from the patient's leukemic cells (Figure 1c). Sequencing analysis of the PCR product revealed that the *SQSTM1* exon 9 was fused to *FGFR1* exon 9 (Figures 1c and d).

A reciprocal *FGFR1*-*SQSTM1* fusion transcript was also detected in using forward *FGFR1*-7F1 (5′-GTAACTCTATCGGACTCTCCC-3′) for exon 7 and reverse *SQSTM1*-11R1 (5′-CGGGGGATGCTTTGAATACTGG-3′) for exon 11 primes (Figures 1c and d).

To identify the chromosomal breakpoints, genomic DNA was amplified by the long-and-accurate PCR method using LA Taq polymerase (Takara Bio). The following primers were used: *FGFR1*-7F1 and *FGFR1*-7F2 (5′-TCTGCATGGTTGACCGTTCTG-3′) for exon 7 outer and inner; *FGFR1*-10R1 and *FGFR1*-10R2 for exon 10 outer and inner; *SQSTM1*-9F1 (5′-GATATCGATGTGGAGCACGGAGGG-3′), *SQSTM1*-9F2 (5′-CTCCAGAGAGTTCCAGCACAGAGG-3′) for exon 9 outer and inner; and *SQSTM1*-10R1 (5′-TGTGGGTACAAGGCAGCTTCC-3′), *SQSTM1*-10R2 (5′-CTTGGCCCTTCGGATTCTGGC-3′) for exon 10 outer and inner, respectively. Genomic fragments corresponding to the der(5) and der(8) chromosomes were amplified by primers around the predicted breakpoints on both chromosomes (Figures 1e and f). The sequence analysis of the PCR products identified the chromosome 5 and 8 breakpoints within *SQSTM1* intron 9 and *FGFR1* intron 9, respectively (data not shown).

As sequence databases contain alternative transcripts for *SQSTM1* coding N-terminal truncated protein (NCBI Reference Sequence: NM_001142298.1 and NM_00114299.1), we performed RT-PCR using primers located in *SQSTM1* alternative exons 1, 2 or 4 and *FGFR1* exon 9 and detected only a chimeric transcript from *SQSTM1* exon 4 coding the full N-terminal end of SQSTM1 (data not shown).

*SQSTM1* at chromosome 5q35 encodes a multifunctional protein that binds ubiquitin and regulates activation of the NF-kB signaling pathway, associating with oxidative stress and autophagy.^6, 7, 8, 9^ Mutations in this gene were reported to cause Paget disease of bone.^10^ The *SQSTM1*-*FGFR1* transcript encodes a predicted chimeric protein of 718 amino acids, containing the N-terminal protein-protein interaction domain, PB1, of SQSTM1 (ref. 11) and the tyrosine kinase domain of FGFR1 (Figure 2). SQSTM1-FGFR1 may induce the constitutive activation of tyrosine kinase by PB1-mediated dimerization, thus leading to the cellular transformation.

Recently, the generation of *SQSTM1-NUP214* and *SQSTM1*-*ALK* fusion genes were reported in one case with adult T-cell acute lymphoblastic leukemia presenting t(5;9)(q35;q34) translocation and two cases with anaplastic lymphoma kinase (ALK)-positive large B-cell lymphoma, respectively.^12, 13, 14^ SQSMT1-ALK was shown to possess transforming activity in 3T3 fibroblasts, probably by the constitutive activation of ALK tyrosine kinase by PB1-mediated dimerization.^13^ To our knowledge, our case is the third instance of hematologic malignancy, in which *SQSTM1* was involved in chromosomal translocation. *SQSTM1* may be a recurrent target of chromosomal aberration.

Our patient, diagnosed as acute myelomonocytic leukemia, lacked typical EMS features, such as myeloproliferation, eosinophilia or lymphadenopathy involved by T-cell proliferation. Multilinage dysplasia indicated the leukemic transformation at early myeloid precursor level, but involvement of the T-cell lineage was not determined. *SQSTM1*-*FGFR1* and/or reciprocal *FGFR1*-*SQSTM1* fusion gene may exert to promote proliferation of immature myelomonocytic cells preferentially. Further investigation is needed to clarify this point.

## Figures and Tables

**Figure 1:**
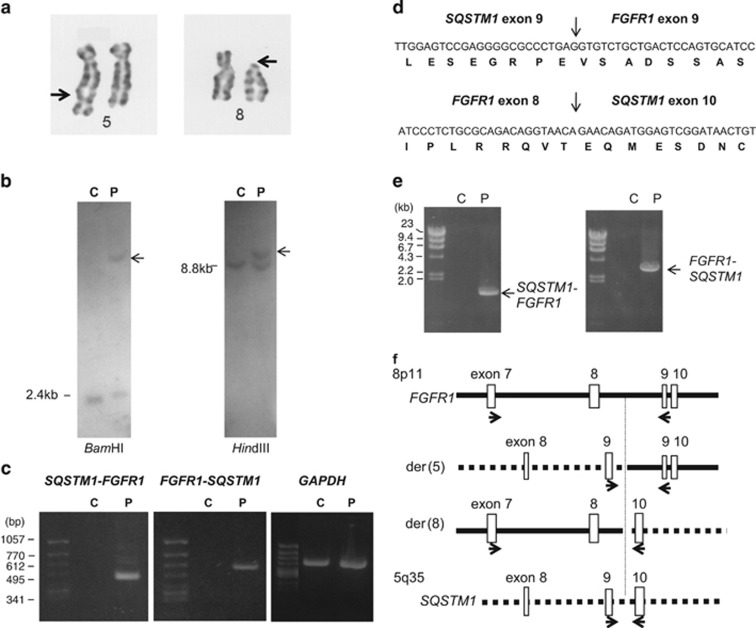
Cytogenetic and molecular analysis. (**a**) Partial karyotyping of patient's bone marrow cells. The derivative chromosome 5 and 8 are shown by arrows. (**b**) Southern blot analysis, presenting rearrangement within *FGFR1* gene. Arrows indicate the abnormal bands. (**c**) RT-PCR detection of the fusion transcripts in the patient. (**d**) Partial sequence of *SQSTM1*-*FGFR1* and reciprocal *FGFR1*-*SQSTM1* fusion cDNA. The amino-acid translations spanning the fusion are shown under the sequence. (**e**) Detection of der(5) and der(8) chromosomes in the patient's leukemic cells. Amplified genomic DNA fragments from the patient's sample are indicated by arrows. (**f**) Genomic organization of *FGFR1* and *SQSTM1*, encompassing the breakpoints. Horizontal arrows indicate primers used in PCR. Lanes C and P represent normal control and the patient's sample, respectively.

**Figure 2:**
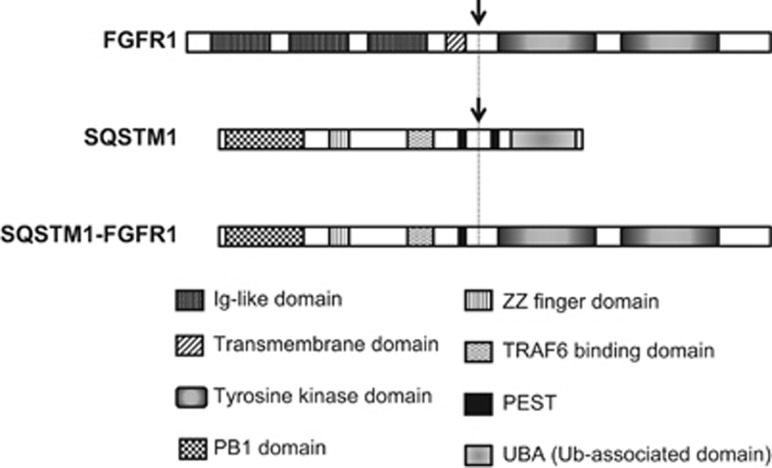
Schematic representation of FGFR1, SQSTM1 and the predicted SQSTM1-FGFR1 fusion protein. Relevant protein domains are shown. The breakpoints within proteins are indicated by arrows.
